# P-433. Impact of Tesamorelin on Cardiovascular Disease Risk Prediction Scores in Phase 3 Studies Treatment Arms: Subanalysis

**DOI:** 10.1093/ofid/ofae631.633

**Published:** 2025-01-29

**Authors:** Steven K Grinspoon, Lindsay Fourman, Takara Stanley, Colleen McGary, David Benkeser, R Brandon Cash

**Affiliations:** Massachusetts General Hospital, Weston, Massachusetts; Massachusetts General Hospital and Harvard Medical School; Massachusetts General Hospital, Weston, Massachusetts; Theratechnologies, Chicago, Illinois; Emory University, Atlanta, GA; Theratechnologies, Inc, Montreal, Quebec, Canada

## Abstract

**Background:**

The risk of cardiovascular disease (CVD) is nearly twice as high for persons with HIV (PWH) as the general population. Excess visceral abdominal fat (EVAF), the key characteristic of central adiposity, has also been associated with an increased risk of CVD in PWH. Tesamorelin, a growth hormone-releasing hormone analog, has previously demonstrated significant reductions in EVAF in two phase 3 randomized controlled trials in PWH. Yet, the impact of the reduction of EVAF from tesamorelin on CVD outcomes has been unknown.

Tesamorelin Treatment Effect on CV Risk Score

**Figure:**
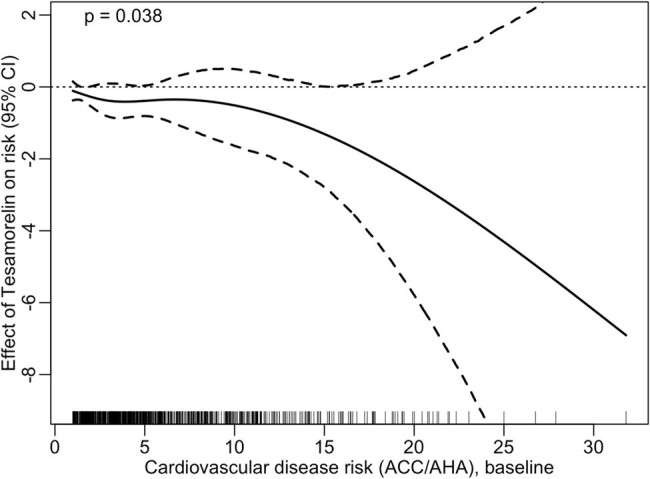

**Methods:**

To assess this impact, combined datasets from the phase 3 studies’ treatment arms were utilized to calculate 10-year atherosclerotic cardiovascular disease (ASCVD) risk scores at baseline and 26 weeks. A mediation analysis was conducted to characterize the significance of treatment impact via intermediate variables that factor into ASCVD risk prediction changes. Modifiable variables included systolic and diastolic blood pressure, total cholesterol (TC), high-density and low-density lipoprotein (HDL, LDL).

The analysis population included 543 subjects randomized to tesamorelin treatment, 86% of which were male and 22% non-white; the median age was 47 years old. The percentage of subjects on lipid-lowering therapies, hypertension treatment, or diabetic were 46%, 37%, and 18%, respectively.

**Results:**

Although the majority had low CVD risk at baseline, 44% had borderline to high CVD risk. Participants in the tesamorelin treatment arm tended toward a modest reduction in 10-year ASCVD risk prediction with estimated decrease in ASCVD risk of -0.40% (95% CI -0.89%, 0.05%). The reduction in CVD risk was relatively larger among subjects with higher CVD risk at baseline (p=0.038 for overall trend among all participants). Reductions in ASCVD risk score were driven predominantly by reductions in TC, independent of lipid lowering therapies.

**Conclusion:**

In summary, this analysis provides evidence that reductions in excess visceral fat with tesamorelin lead to a significant reduction in forecasted CVD risk in PWH, resulting from reduction in TC even among a group heavily treated with lipid-lowering therapy. Given the increasing prevalence of obesity and central adiposity in PWH, more attention should be given to targeting visceral fat when considering CVD risk management.

**Disclosures:**

**Steven K. Grinspoon, MD**, Gilead: Grant/Research Support|Kowa: Grant/Research Support|Theratechnologies: Advisor/Consultant|Viiv: Grant/Research Support **Lindsay Fourman, MD**, Chiesi Farmaceutici: Advisor/Consultant|Chiesi Farmaceutici: Grant/Research Support|Theratechnologies: Advisor/Consultant **Takara Stanley, MD**, Pfizer: Grant/Research Support **Colleen McGary, PhD**, Theratechnologies: Employee **R Brandon Cash, PharmD**, Theratechnologies: Employee

